# Estimation of the number of single-photon emitters for multiple fluorophores with the same spectral signature

**DOI:** 10.1116/5.0162501

**Published:** 2023-11-14

**Authors:** Wenchao Li, Shuo Li, Timothy C. Brown, Qiang Sun, Xuezhi Wang, Vladislav V. Yakovlev, Allison Kealy, Bill Moran, Andrew D. Greentree

**Affiliations:** 1 School of Science, RMIT University, Melbourne, VIC 3001, Australia; 2 ARC Centre of Excellence for Nanoscale BioPhotonics, RMIT University, Melbourne, VIC 3001, Australia; 3 School of Mathematics, Monash University, Melbourne, VIC 3800, Australia; 4 Department of Biomedical Engineering, Texas A&M University, College Station, Texas 77843, USA; 5 School of Engineering, RMIT University, Melbourne, VIC 3001, Australia; 6 Department of Electrical and Electronic Engineering, University of Melbourne, VIC 3010, Australia

## Abstract

Fluorescence microscopy is of vital importance for understanding biological function. However, most fluorescence experiments are only qualitative inasmuch as the absolute number of fluorescent particles can often not be determined. Additionally, conventional approaches to measuring fluorescence intensity cannot distinguish between two or more fluorophores that are excited and emit in the same spectral window, as only the total intensity in a spectral window can be obtained. Here we show that, by using photon number resolving experiments, we are able to determine the number of emitters and their probability of emission for a number of different species, all with the same measured spectral signature. We illustrate our ideas by showing the determination of the number of emitters per species and the probability of photon collection from that species, for one, two and three otherwise unresolvable fluorophores. The convolution binomial model is presented to represent the counted photons emitted by multiple species. Then, the expectation-maximization (EM) algorithm is used to match the measured photon counts to the expected convolution binomial distribution function. In applying the EM algorithm, to leverage the problem of being trapped in a sub-optimal solution, the moment method is introduced to yield an initial guess for the EM algorithm. Additionally, the associated Cramér–Rao lower bound is derived and compared with the simulation results.

## I. INTRODUCTION

Understanding the complex biological function often requires precise localization of biological molecules in space and time to better understand their interactions and relevant chemical reactions and transformations. Cryogenic electron microscopy is capable of defining positions of atoms in complex molecules with angstrom accuracy^1^ but is often limited in capturing complex interactions of molecules in dynamic biological environment. Fluorescence and Raman microspectroscopies can provide structural and functional information about biological molecules, though special measures are needed to extend the spatial resolution of optical imaging beyond the traditional diffraction limit defined by the excitation wavelength of light. The new generation of optical imaging methods based on super-resolution optical imaging, for which the Nobel Prize in Chemistry was awarded in 2014, is gradually being adopted by the research community, and these techniques are now indispensable for fundamental understanding of biological function at the molecular level. However, many biological molecules are performing their function not along but in a coherent ensemble with other molecules. A typical example of such synergistic interaction is the electron-transfer complex in a mitochondrial membrane, where several cytochromes are involved in the ultimate production of ATP molecules that serve as a major energy fuel for a cell. To understand the function of a mitochondrion and to assess its ability to efficiently produce ATP molecules, one needs to quantify the number of such cytochromes in a membrane, which is close to impossible using conventional methods. There are several challenges which are related to the size of the focal spot and internal dynamics of mitochondria, and these make it difficult to localize those different cytochromes within the membrane. Traditional approaches based on classical photon statistics have significant limitations, because of either their invasive nature, such as induced photobleaching, which affects the electronic structure of biological fluorophores and amends its function, or complexity of signal collection and analysis.^2^ On the other hand, quantum spectroscopy based on the quantum statistics of detecting photons using photon resolved detectors and cameras^3,4^ (see also qCMOS by Hamamatsu^5^) as we have shown recently^6^ makes it feasible to count individual emitters in a focal volume.

Techniques of fluorescence microscopy have become among the most used techniques for understanding biological function.^7–12^ These typically involve measurement of the uptake of functionalized fluorophores, or observation of the expression of fluorescent proteins in response to some stimulus.^8–12^ Fluorescence experiments are usually *qualitative* or, at most, relative, as the total number of fluorophores is often not observable because a conventional intensity measurement is unable to distinguish a few bright emitters from many dim emitters.^8^ Moreover, if the fluorophores are excited and emit in the same spectral windows, then they may be impossible to distinguish with intensity only measurements. In Ref. 13, the average number of emitters in each species and the brightness ratio between multiple species are investigated and evaluated using high-order image correlation spectroscopy.

Quantum correlation techniques use two-photon and higher coincidences to gain information about an incoming light field. As such, they have found application in imaging and localization, e.g.,^14–19^ characterization^6,20^ as well as in the identification of non-classical states of light.^21,22^ The traditional method of measuring coincidences is through the Hanbury Brown and Twiss experiment,^23^ where single photon avalanche detectors (SPADs) monitor the light received via a beamsplitter, or sequence of beamsplitters in the case of higher order coincidences. Sometimes, systems that monitor higher-order coincidences in this fashion are referred to as photon number resolving detectors (PNRD) (see, for example, Ref. 22) although here we reserve this terminology for devices that are inherently photon resolving such as those we mention above. A comparison of high-order Hanbury Brown and Twiss with photon number resolving detectors can be found in Ref. 6.

Earlier, we showed that the problem of quantitative determination of the number of emitters and the probability of photon detection could be solved for a single species of emitters with assumed identical properties.^6^ Our approach there used the binomial distribution of the number of photons emitted in a pulsed fluorescence experiment. By considering photon number resolving detectors (PNRD),^4,24–31^ we showed that the distribution of photons arriving in each detected pulse is uniquely specified by the number of emitters and the photon detection probability, so that these parameters can be determined with some confidence given a particular measurement record.

Here, we show that PNRD detection techniques can, in principle, be used to discriminate between an arbitrary number of fluorescent species. We assume that each species is defined by two parameters, the number of emitters, *M*, and the probability of detection *p*, which is assumed to be constant for each member of the species, for instance, by each species having a different transition dipole moment. We assume no further ability to distinguish the species. The determination of multiple species is a highly non-trivial extension of our earlier work.^6^ The additional complexity arises due to the multi-dimensional state space which leads to multiple local minima, confounding convergence to the ground truth. To mitigate these issues, we introduce a two-stage estimator, which we find improves convergence significantly. Our treatment assumes that an upper bound on number of species is known *a priori* (some species could have either *p* = 0 or *M* = 0), but for computational simplicity here we will assume that the number of distinct species is known.

This paper is organized as follows. We first introduce the measurement model and maximum likelihood estimation (MLE) approach. We then discuss how the expectation-maximization (EM) technique is applied. Finally, we present simulations and Cramér–Rao lower bounds (CRLB) for the cases of one, two, and three species.

## II. MEASUREMENT MODEL AND MLE

**Fig. 1.**
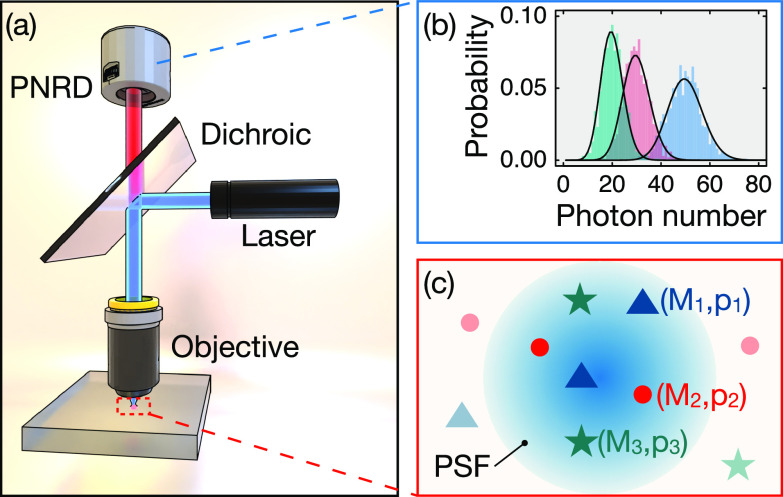
(a) Schematic of using PNRD to discriminate three species of emitters. The excitation laser (blue beam) passes through the dichroic mirror and focuses on three species (*M*_1_, *p*_1_), (*M*_2_, *p*_2_), and (*M*_3_, *p*_3_) under the diffraction limit locating within one Gaussian focal spot in (c). The emitting light (red beam) is detected by PNRD, and the synthetic photon number resolving signal is shown in (b) as bar charts in three colors. With the measurement time increases, the probability of detecting the photon numbers would follow Poisson distribution, shown as the black smooth curves. The area underneath each Poisson curve or bar chart is always one, indicating that in a practical measurement the photon counts or the intensity from each species is the same; therefore, they cannot be identified by conventional intensity-only based microscopy. However, they generate distinguishing PNRD signals, building on which we introduce the MLE approach to tell them apart.

We consider a fluorescence experiment where there are *m* distinct fluorophores (species); species *j* has *M_j_* emitters with probability of photon detection from each member of that species *p_j_*. A single excitation source (e.g., laser through microscope objective) excites the sample, and the fluorescence photons are collected by a PNRD. Schematic with three species emitters is shown in Fig. 1.

Due to the numerical complexity of the current scheme, we make a simplifying assumption that each emitter species has the same detection probability per emitter. The point spread function of a microscope system means that as the emitters' positions vary, the collection efficiency would also vary. For some spatial distribution of the emitters, then the ith emitter from group *j* will have collection probability $p_{i,j}=p_{i,j}(r_{i,j})$, where $r_{i,j}$ is the distance of the emitter from the focal point of the objective. If these detection probabilities overlap, then without additional information (for example an *ansatz* describing the spatial distribution of the emitters) it will not be possible to distinguish bright emitters with large *r* from less bright emitters with small *r*, which in turn implies that estimates of the *M_j_* will be problematic. These restrictions mean that we require the emitters to be confined to a region much smaller than the point spread function, or to use uniform illumination and an integrating sphere to collect the emission.

This experiment is repeated a large number of times to build up statistics about the system. The number of fluorescence photons detected from each pulse, *y*, is counted at the outputs of the emitters; this is modeled as the sum of *m* random variables sampled from binomial distributions with parameters $[M_{j},p_{j}],\;j=1,\ldots ,m$, i.e.,

$$Y=\sum_{j=1}^{m}Y_{j},\;\text{where}\;Y_{j}∼B(M_{j},p_{j}).$$ (1)

The probability mass function (PMF) of *Y* = *y*, y=0,…,M and $M=\sum_{i=1}^{m}M_{i}$, given parameters $\theta =\left[M_{1},p_{1},M_{2},p_{2},\ldots ,M_{m},p_{m}\right]$ can be seen, by the law of total probability, to be a convolution of *m* binomial distributions, $pr_{j}(y_{j}|M_{j},p_{j})$ for j=1,…,m and $y_{j}=0,1,\ldots ,M_{j}$, and so can be derived as

$$\begin{array}{c} pr(Y=y|\theta )=\sum_{y\in Y_{y}}\left(\prod_{j=1}^{m}\frac{M_{j}}{(M_{j}-y_{j})!y_{j}!}p_{j}^{M_{j}}{\left(1-p_{j}\right)}^{M_{j}-y_{j}}\right)\\ \overset{△}{=}\sum_{y\in Y_{y}}\left(\prod_{j=1}^{m}pr_{j}(y_{j}|M_{j},p_{j})\right), \end{array}$$ (2)

where $y=\left[y_{1},\ldots ,y_{m}\right]$ and

$$Y_{y}=\left\{\left[y_{1},\ldots ,y_{m}\right]|\sum_{j=1}^{m}y_{j}=y,y_{j}\in \left[0,1,2,\ldots ,M_{j}\right]\right\}.$$

$Y_{y}$ is actually the collection of partition of the integer *y* into exactly *m* parts. It should be noted that background noise, such as stray light, objective autofluorescence, and camera noise are not considered in our model. These noise sources may be modeled by Poisson or mixed Poisson Gaussian model,^32^ and researchers have proposed some methods to eliminate or compress these noise.^33–35^ Although treating this noise is practically important, it represents a significant complication to our model, greatly increasing the necessary space to explore and thereby distracting from the main goals of this paper. We, therefore, omit background noise in the model (2), leaving this as a future problem.

The fundamental problem of interest is to estimate θ based on the measurements. We show that this problem can be formulated as an MLE. In Ref. 36, it is shown that the probability of the sum of *m* independent integer-valued random variables (not necessarily identically distributed), i.e., pr(Y=y|θ), may be calculated using a recurrence relation. Furthermore, the PMF of (1) can be approximated by a Gaussian distribution, $N\left[\sum_{j=1}^{m}Mp_{j},\sum_{j=1}^{m}M_{j}p_{j}(1-p_{j})\right]$. In Refs. 37–39, other more accurate approximation methods, such as saddlepoint approximation, Kolmogorov approximation, or Krawtchouk polynomial approximation, are provided. Since all the mentioned methods are either in the form of a recurrence formula or otherwise have no closed form, they cannot be directly used in deriving the MLE for θ. Accordingly, we use Eq. (2) for investigating the MLE.

In each experiment, we record the peak corresponding photon number from the PNRD signal as *i*, i=0,…,N and N<=M. We count the occurrence of *i* in a series of experiments as *C_i_*; then, the data from a series of experiments can be given by the frequency distribution $\left[C_{0},C_{1},\ldots ,C_{N}\right]$, and $\nu =\sum_{i=0}^{N}C_{i}$ is the total number of experiments. The log-likelihood function can then be expressed as

$$\begin{array}{c} \ell (C_{0},\ldots ,C_{N}|\theta )=\sum_{i=0}^{N}C_{i}\;\text{log}\;\sum_{y\in Y_{i}}\left(\prod_{j=1}^{m}pr_{j}(y_{j}|M_{j},p_{j})\right)\\ \overset{△}{=}\sum_{i=0}^{N}C_{i}\;\text{log}\;L(y|\theta ), \end{array}$$ (3)

where $L(y|\theta )=\sum_{y\in Y_{i}}\left[\prod_{j=1}^{m}pr_{j}(y_{j}|M_{j},p_{j})\right]$; similar to $Y_{y},\;Y_{i}$ is the collection of partition of the integer *i* into *m* parts. Furthermore, we assume that $f(y|\theta )=\prod_{j=1}^{m}pr_{j}(y_{j}|M_{j},p_{j})$ and therefore $L(y|\theta )=\sum_{y\in Y_{i}}f(y|\theta )$.

The MLE of $\theta ,\;\hat{\theta }=[{\hat{M}}_{1},{\hat{p}}_{1},\ldots ,{\hat{M}}_{m},{\hat{p}}_{m}]$, is

$$\hat{\theta }=\text{arg}\underset{\theta \in \times_{j=1}^{m}\left({\mathbb{Z}}^{+}\times [0,1]\right)}{\text{max}}\ell (C_{0},\ldots ,C_{N}|\theta ),$$ (4)

where × is the Cartesian product. From (2) to (4), the underlying estimation problem is formulated as a parameterized MLE problem; that is, seeking the set of parameters θ in the parameter space which yield maximum likelihood $\ell (C_{0},\ldots ,C_{N}|\theta )$ based on the observations.

The solution to (4) when *m* = 1 was provided in Ref. 6, where the MLE is proved to be an effective estimator and the associated CRLB is derived. However, when *m* > 1, solving (4) directly is very inefficient and even computationally impossible since the dimension of the problem, i.e., the number of the parameters to be estimated, is 2*m*. This rules out grid-based methods to find the MLE. With the increasing number of parameters, the existence of multiple local extrema confounds most optimization methods for finding the global extremum.

**Fig. 2.**
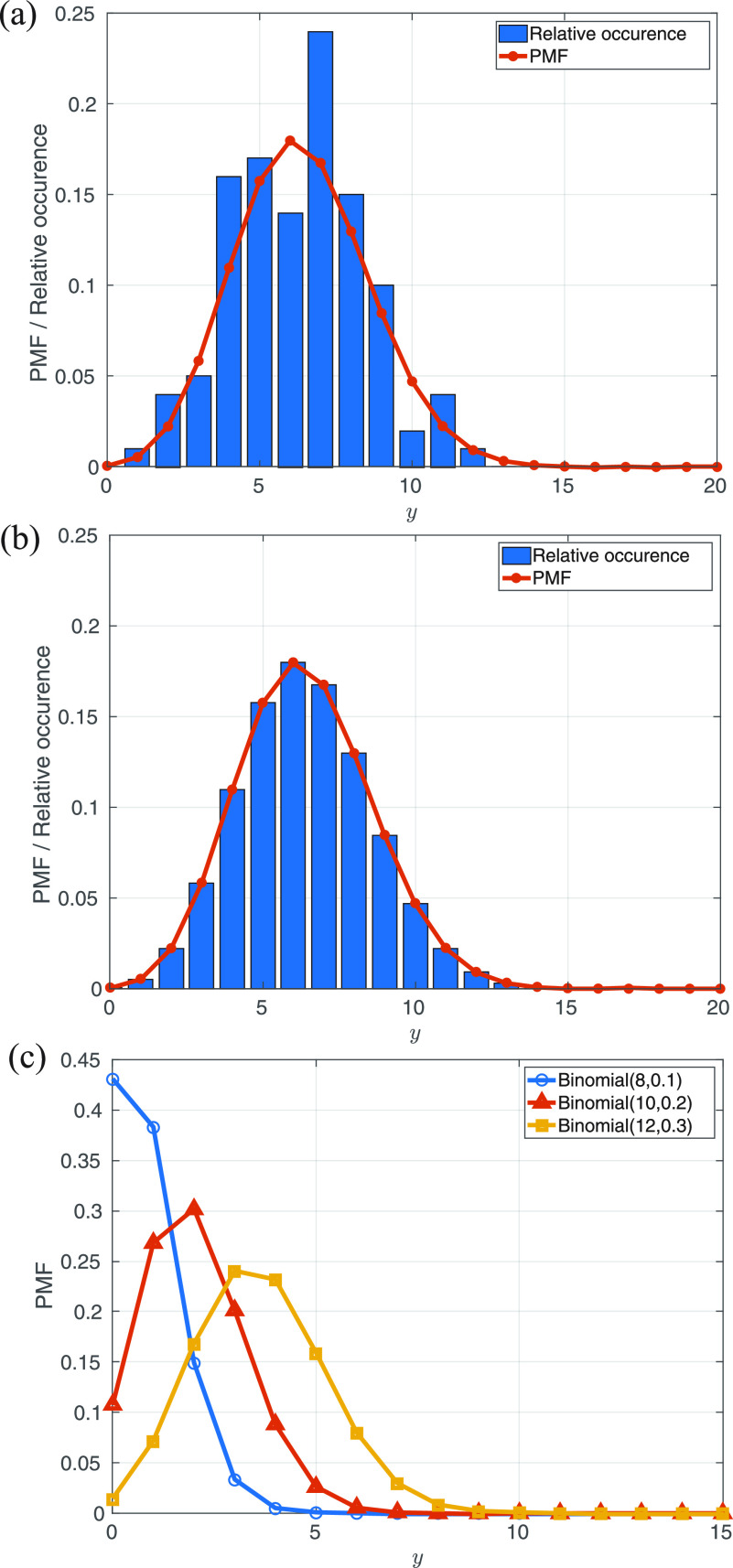
The comparison between the relative occurrence obtained from synthetic measurement from photons with different *ν*, (a) *ν* = 100 and (b) ν=1e7, where θ=[8,0.1,10,0.2,12,0.3]. (c) shows the binomial PMF for each species. It can be seen that the histogram obtained from the synthetic data converges to the expected distribution of photons with the increasing of the *ν.*

To investigate the determination of θ, we generated synthetic data using the PMF, Eq. (2), with total number of experiments *ν*. These synthetic data yield a histogram of events, as illustrated in Fig. 2. The synthetic data were generated on the basis of *ν* = 100 experiments, with parameter θ=[8,0.1,10,0.2,12,0.3]. Also shown in Fig. 2(a), the expected PMF given by Eq. (2), the histogram of synthetic data when *ν* = 100. As the number of experiments increases, the synthetic data should converge to the expected PMF as shown in Fig. 2(b).

**Remark 1.** *In the estimation of the parameters of fluorescent species, one may be more interested in the number of emitters of each species rather than the probabilities, so that, ideally, more consideration should be given to that problem. However, in the estimation of* $\left[M_{j},p_{j}\right]$*, their accuracies are correlated; that is, reduced accuracy of estimation of p_j_ will lead to a worse estimation performance for M_j_. Additionally, because of the integral nature of M_j_, the estimator* ${\hat{M}}_{j}$ *may be well away from ground truth, corresponding to a small change of* ${\hat{p}}_{j}$*. Accordingly, we will not focus on a particular parameter in what follows.*

## III. EXPECTATION-MAXIMIZATION ALGORITHM

The EM algorithm is an effective method for finding the MLE (or local extremum of the likelihood) iteratively by a simplification of a complicated likelihood function. By carefully choosing the initial guess of the EM, the MLE is approached with high probability.

In this section, the likelihood function $\ell (C_{0},\ldots ,C_{N}|\theta )$, i.e., (3), is firstly reformulated and simplified to an equivalent problem under the EM framework using the sum-of-log-of-sums method.^40^ As a result, the 2*m* dimension MLE problem is converted into *m* independent two-dimension optimization problems for which local extrema can be found iteratively, see (12).

Since the likelihood has many local minima, the initial guess has a significant impact on the final estimate of the EM algorithm, i.e., the EM algorithm may converge to a local critical point with an “incorrect” initial guess. It should be noted that EM becomes more sensitive to the initial guess with increasing *m*, as the number of local extrema increases for larger *m*. Seeding the EM algorithm with a limited number of random initial guesses typically provides convergence to the MLE when *m* = 2, but the number of initial seeds becomes unacceptably large when *m* > 2. To overcome this problem, we combined the moment estimator with EM algorithm; that is, the results of moment estimator are used as the initial guesses.

**Remark 2.** *We mention that it is non-trivial to find the global optimum, i.e., exact MLE, for the likelihood function* (3) *since the likelihood function is highly complex* and *exhibits multiple local minima. Some existing optimization methods, such as Annealing,*^41^ *particle swarm optimization*^42^ and *stochastic gradient descent,*^43,44^ *are available to find the global optimum for simple cost function, but there is no guarantee that these method can find the global optimum for highly nonlinear functions having multiple local optimum such as found here in the case of multiple species.*

### A. Reformulating the likelihood function for EM algorithm

We introduce an intermediate variable ${\hat{\theta }}^{\left\langle s\right\rangle }$ that is the estimate of θ at the *s*th iteration of the EM algorithm. Given ${\hat{\theta }}^{\left\langle s\right\rangle }$, from (3), we have

$$\begin{array}{c} \text{log}\;L(y|\theta )-\text{log}\;L(y|{\hat{\theta }}^{\left\langle s\right\rangle })\\ =\text{log}\;\left[\frac{\sum_{y\in Y_{i}}\frac{f(y|\theta )}{f(y|{\hat{\theta }}^{\left\langle s\right\rangle })}f(y|{\hat{\theta }}^{\left\langle s\right\rangle })}{L(y|{\hat{\theta }}^{\left\langle s\right\rangle })}\right] \end{array}$$ (5)

$$\geq \sum_{y\in Y_{i}}w_{y}^{\left\langle s\right\rangle }\;\text{log}\;\left[\frac{f(y|\theta )}{f(y|{\hat{\theta }}^{\left\langle s\right\rangle })}\right]$$ (6)

$$=\sum_{y\in Y_{i}}w_{y}^{\left\langle s\right\rangle }\;\text{log}\;f(y|\theta )-\sum_{y\in Y_{i}}w_{y}^{\left\langle s\right\rangle }\;\text{log}\;f(y|{\hat{\theta }}^{\left\langle s\right\rangle })$$ (7)

$$=\tilde{Q}(\theta |{\hat{\theta }}^{\left\langle s\right\rangle })-\tilde{Q}({\hat{\theta }}^{\left\langle s\right\rangle }|{\hat{\theta }}^{\left\langle s\right\rangle }),$$ (8)

where (6) follows by Jensen's inequality,^40^ with $w_{y}^{\left\langle s\right\rangle }=f(y|{\hat{\theta }}^{\left\langle s\right\rangle })/L(y|{\hat{\theta }}^{\left\langle s\right\rangle })$ and $\sum_{y\in Y_{i}}w_{y}^{\left\langle s\right\rangle }=1$.

In a similar way, we consider the joint log-likelihood function $\ell (C_{0},\ldots ,C_{N}|\theta )$. In this case, the auxiliary function is

$$\begin{array}{c} Q(\theta |{\hat{\theta }}^{\left\langle s\right\rangle })=\sum_{i=0}^{N}C_{i}\sum_{y\in Y_{i}}w_{y}^{\left\langle s\right\rangle }\;\text{log}\;f(y|\theta )\\ =\sum_{i=0}^{N}C_{i}\sum_{y\in Y_{i}}w_{y}^{\left\langle s\right\rangle }\sum_{j=1}^{m}\;\text{log}\;g(y_{j}|\theta_{j}), \end{array}$$ (9)

where f(y|θ) is defined in (3) and

$$g(y_{j}|\theta_{j})=\frac{M_{j}}{(M_{j}-y_{j})!y_{j}!}p_{j}^{M_{j}-y_{j}}{\left(1-p_{j}\right)}^{y_{j}}.$$ (10)

One can find a local extremum of log L(y|θ) by maximizing $\tilde{Q}(\theta |{\hat{\theta }}^{\left\langle s\right\rangle })$ iteratively.^45^ The problem then becomes to maximize $Q(\theta |{\hat{\theta }}^{\left\langle s\right\rangle })$ over θ, i.e.,

$${\hat{\theta }}^{\left\langle s+1\right\rangle }=\left\{{\hat{\theta }}_{1}^{\left\langle s+1\right\rangle },\ldots ,{\hat{\theta }}_{m}^{\left\langle s+1\right\rangle }\right\}=\text{arg}\underset{y\in \times_{j=1}^{m}\left([0,1]\times {\mathbb{Z}}^{+}\right)}{\text{max}}Q(\theta |{\hat{\theta }}^{\left\langle s\right\rangle }).$$ (11)

Since, $\text{log}\;g(y_{j}|\theta_{j}),\;j=1,2,\ldots ,m$ are independent with respect to $\theta_{j^{-}}$, where $j^{-}=\{1,\ldots ,m\}\setminus \{j\}$, (11) can be rewritten as

$$\begin{array}{c} {\hat{\theta }}^{\left\langle s+1\right\rangle }=\{{\hat{\theta }}_{1}^{\left\langle s+1\right\rangle }=\text{arg}\underset{\theta_{1}\in [0,1]\times {\mathbb{Z}}^{+}}{\text{max}}\sum_{i=0}^{N}C_{i}\sum_{y\in Y_{i}}w_{y}^{\left\langle s\right\rangle }\;\text{log}\;g(y_{1}|\theta_{1}),\;\ldots ,\\ {\hat{\theta }}_{m}^{\left\langle s+1\right\rangle }=\text{arg}\underset{\theta_{m}\in [0,1]\times {\mathbb{Z}}^{+}}{\text{max}}\sum_{i=0}^{N}C_{i}\sum_{y\in Y_{i}}w_{y}^{\left\langle s\right\rangle }\;\text{log}\;g(y_{m}|\theta_{m})\}\\ =\{{\hat{\theta }}_{1}^{\left\langle s+1\right\rangle }=\text{arg}\underset{\theta_{1}\in [0,1]\times {\mathbb{Z}}^{+}}{\text{max}}Q_{1}\left(\theta_{1}|{\hat{\theta }}^{\left\langle s\right\rangle }\right),\ldots ,\\ {\hat{\theta }}_{m}^{\left\langle s+1\right\rangle }=\text{arg}\underset{\theta_{m}\in [0,1]\times {\mathbb{Z}}^{+}}{\text{max}}Q_{m}\left(\theta_{m}|{\hat{\theta }}^{\left\langle s\right\rangle }\right)\}. \end{array}$$ (12)

For j∈{1,…,m}, we have

$${\hat{\theta }}_{j}^{\left\langle s+1\right\rangle }=\text{arg}\underset{\theta_{j}\in [0,1]\times {\mathbb{Z}}^{+}}{\text{max}}Q_{j}\left(\theta_{j}|{\hat{\theta }}^{\left\langle s\right\rangle }\right).$$

Then, ${\hat{p}}_{j}^{\left\langle s+1\right\rangle }$ can be found via

$$\frac{\partial }{\partial p_{j}}Q_{j}\left(\theta_{j}|{\hat{\theta }}^{\left\langle s\right\rangle }\right)=0\;\Rightarrow \;{\hat{p}}_{j}^{\left\langle s+1\right\rangle }=\frac{\sum_{i=0}^{N}C_{i}\sum_{y\in Y_{i}}w_{y}^{\left\langle s\right\rangle }y_{j}}{{\hat{M}}_{j}^{\left\langle s\right\rangle }\nu }.$$ (13)

Substituting ${\hat{p}}_{j}^{\left\langle s+1\right\rangle }$ into *j*th term of (12), we obtain

$${\hat{\theta }}_{j}^{\left\langle s+1\right\rangle }=\left\{{\hat{M}}_{j}^{\left\langle s+1\right\rangle },\;{\hat{p}}_{j}^{\left\langle s+1\right\rangle }\right\},$$ (14)

where

$${\hat{M}}_{j}^{\left\langle s+1\right\rangle }=\text{arg}\underset{M_{j}\in {\mathbb{Z}}^{+}}{\text{max}}Q_{j}\left(M_{j}|{\hat{M}}_{j}^{\left\langle s\right\rangle },{\hat{p}}_{j}^{\left\langle s+1\right\rangle }\right).$$ (15)

Now, the EM algorithm can be implemented using an initial guess ${\hat{\theta }}_{j}^{\left\langle 0\right\rangle }$ for j=1,…,m and the (local) estimate can be obtained until ${\hat{\theta }}_{j}^{\left\langle s\right\rangle }$ converges. The structure of the EM algorithm is listed in Algorithm 1.

**Algorithm 1.** The EM algorithm to estimate θ.

**Data:** $C_{0},\ldots ,C_{N}$
**Result:** $\hat{\theta }\leftarrow [{\hat{M}}_{1},{\hat{p}}_{1},\ldots ,{\hat{M}}_{m},\ldots ,{\hat{p}}_{m}]$ s←0;
Choose initial guesses $\left[{\hat{M}}_{1}^{\left\langle s\right\rangle },\ldots ,{\hat{M}}_{m}^{\left\langle s\right\rangle }\right]$ and $\left[{\hat{p}}_{1}^{\left\langle s\right\rangle },\ldots ,{\hat{p}}_{m}^{\left\langle s\right\rangle }\right]$
**repeat**
s←s+1;
Calculate ${\hat{p}}_{j}^{\left\langle s\right\rangle }$ using (13) and (14) for j=1,…,m;
**until** *converge*;
$\hat{\theta }\leftarrow [{\hat{M}}_{1}^{\left\langle s\right\rangle },p_{1}^{\left\langle s\right\rangle },\ldots ,{\hat{M}}_{m}^{\left\langle s\right\rangle },p_{m}^{\left\langle s\right\rangle }]$;

### B. Choice of the initial guess for the EM algorithm

In the EM algorithm, the log likelihood is guaranteed to increase at each EM iteration, and it converges to a maximum of the likelihood under mild conditions.^45^ However, it is not guaranteed to be the global optimum.

As an “always improving” algorithm,^46^ EM is, of course, sensitive to the initial guess of θ, i.e., ${\hat{\theta }}^{\left\langle 0\right\rangle }$ when the likelihood function contains multiple critical points. We observe that the number of local critical points increases dramatically with the increasing number of species.

#### 1. Choosing initial guesses for $\left[M_{1},\ldots ,M_{m}\right]$

When *m* > 1, (14) may converge to a local minimum that is not the MLE. To relieve this problem, a search procedure can be adopted into the EM algorithm. In (14), it can be observed that $p_{j}^{\left\langle s+1\right\rangle }$ is updated at each iteration step using ${\hat{M}}_{j}^{\left\langle s\right\rangle }$. By taking into account that $p_{j}^{\left\langle s+1\right\rangle }$ has a better chance to converge to *p_j_* when ${\hat{M}}_{j}^{\left\langle s\right\rangle }$ converges to *M_j_*, one can fix ${\hat{M}}_{j}^{\left\langle s\right\rangle }=M_{j},\;\forall s$ and j=1,…,m, in (14) and then find the optimized ${\hat{p}}_{j}^{\left\langle s\right\rangle }$ given $\left[M_{1},\ldots ,M_{m}\right]$ iteratively.

In practice, $\left[M_{1},\ldots ,M_{m}\right]$ is unknown and to be estimated. However, since $M_{1},\;\ldots ,\;M_{m}$ are (bounded) integers so that their possible values are finite and listable by enumeration. Suppose that $M_{j}\leq M,\;\forall j$ and $M\in {\mathbb{Z}}^{+}$, then a set, $M=\{M_{1},\ldots ,M_{L}\}$, containing all possible combinations for $\left[M_{1},\ldots ,M_{m}\right]$ can be constructed by *m*-combinations from the integer set {1,2,…,M} without repetition, order does not matter and it can be verified that $L=\left(\begin{array}{c} M\\ n \end{array}\right)$. In other words, $M_{l},\;l=1,\ldots ,L$, corresponds to a possible solution (combination) to $\left[M_{1},\ldots ,M_{m}\right]$. The EM algorithm is, therefore, implemented *L* times. At the *l*th implementation, the guess $\left[{\hat{M}}_{1}^{\left\langle 0\right\rangle },\ldots ,{\hat{M}}_{m}^{\left\langle 0\right\rangle }\right]$ is chosen to be $M_{l}$ and fixed for all *s*. For simplicity, we denote $\left[{\hat{M}}_{m,l},\ldots ,{\hat{M}}_{m,l}\right]$ as the guess of $\left[M_{1},\ldots ,M_{m}\right]$ at *l*th implementation of EM algorithm.

#### 2. Choice of initial guesses for $\left[p_{1},\ldots ,p_{m}\right]$

To obtain initial guesses for the estimator of $\left[p_{1},\ldots ,p_{m}\right]$, we use the estimates from the moment estimator. By using the data, it is straightforward to calculate the sample mean, ${\hat{\mu }}_{1}=\sum_{i=0}^{N}\left(C_{i}/\nu \right)i$, and *k*th sample central moments, ${\hat{\mu }}_{k}=\sum_{i=0}^{N}\left(C_{i}/\nu \right){\left(i-{\hat{\mu }}_{1}\right)}^{k}$ for *k* > 1. The associated population mean and central moments are $\mu_{1}=E[x]$ and $\mu_{k}=E[{\left(x-\mu_{1}\right)}^{k}]$ for *k* > 1. Then, the moment estimator, ${\hat{\theta }}^{\text{mom}}$, can be obtained by solving ${\hat{\mu }}_{i}=\mu_{i}$ for i=1,…,m, and this can be used to seed the EM algorithm: ${\hat{\theta }}^{\left\langle 0\right\rangle }={\hat{\theta }}^{\text{mom}}$. Since we may find multiple ${\hat{\theta }}^{\text{mom}}$ from the moment estimator, the EM can be run in parallel with different initial guesses.

As an example, the moments *μ_i_* of the sum of *m* = 4 binomial distributed random variables are^38^

$$\{\begin{array}{c} \mu_{1}=\sum_{j=1}^{m}M_{j}p_{j},\\ \mu_{2}=\sum_{j=1}^{m}\left(1-p_{j}\right)M_{j}p_{j},\\ \mu_{3}=\sum_{j=1}^{m}\left(1-p_{j}\right)\left(1-2p_{j}\right)M_{j}p_{j},\\ \mu_{4}=\sum_{j=1}^{m}M_{j}p_{j}\left(1-p_{j}\right)\left(1+\left(3M_{j}-6\right)\left(1-p_{j}\right)p_{j}\right), \end{array}$$

which can be simplified to

$$\{\begin{array}{c} \sum_{j=1}^{m}M_{j}p_{j}=\mu_{1},\\ \sum_{j=1}^{m}M_{j}p_{j}^{2}=\mu_{1}-\mu_{2},\\ \sum_{j=1}^{m}M_{j}p_{j}^{3}=\frac{1}{2}\left(2\mu_{1}-3\mu_{2}+\mu_{3}\right),\\ \sum_{j=1}^{m}M_{j}p_{j}^{4}=\frac{1}{6}\left(6\mu_{1}-11\mu_{2}+3\mu_{2}^{2}+6\mu_{3}-\mu_{4}\right). \end{array}$$ (16)

Applying the method of moments, we replace $\left[M_{1},\ldots ,M_{m}\right]$ by $\left[{\hat{M}}_{1,l},\ldots ,{\hat{M}}_{m,l}\right]$ and $\left[\mu_{1},\ldots ,\mu_{m}\right]$ by $\left[{\hat{\mu }}_{1,l},\ldots ,{\hat{\mu }}_{m,l}\right]$ in (16) and find the real solutions $\left[{\hat{p}}_{1,l},\ldots ,{\hat{p}}_{m,l}\right]$ to provide the initial guess of $\left[p_{1},\ldots ,p_{m}\right]$ at the *l*th implementation of EM algorithm. After obtaining multiple candidate estimates with different initial guesses, the estimated MLE among these candidates is the one with the largest value of the likelihood function (3). The algorithm is summarized in Algorithm 2.

**Algorithm 2.** The algorithm to estimate θ combining searching strategy.

**Data:** $C_{0},\ldots ,C_{N}$
**Result:** $\hat{\theta }\leftarrow [{\hat{M}}_{1},{\hat{p}}_{1},\ldots ,{\hat{M}}_{m},\ldots ,{\hat{p}}_{m}]$
$\left\{M_{1},\ldots ,M_{L}\}\leftarrow \text{nchoosek}(N,m\right)$;
k←1;
**for** l=1:L **do**
$\text{sol}=\text{solve}\left(\sum_{j=1}^{m}{\hat{M}}_{j,l}p_{j}^{i}={\hat{\mu }}_{i},\;\;\;i=1,\ldots ,m\right)$;
**if** *sol is real* **then**
s←0;
${\hat{p}}_{1,l}^{\left\langle s\right\rangle },\ldots ,{\hat{p}}_{m,l}^{\left\langle s\right\rangle }\leftarrow \text{sol}$;
**repeat**
s←s+1;
Find ${\hat{p}}_{j,l}^{\left\langle s\right\rangle }$ using (14) by replacing ${\hat{M}}_{j}^{\left\langle s\right\rangle }$ with ${\hat{M}}_{j,l}$;
**until** *converge*;
${\hat{\theta }}_{k}=[{\hat{M}}_{1,l},{\hat{p}}_{1,l}^{\left\langle s\right\rangle },\ldots ,{\hat{M}}_{m,l},{\hat{p}}_{m,l}^{\left\langle s\right\rangle }]$;
$e_{k}=\ell ({\hat{\theta }}_{l}|C_{0},\ldots ,C_{N})$;
k←k+1;
$I\leftarrow \text{arg}\underset{i}{\text{max}}\;\{e_{i}\}$;
$\hat{\theta }\leftarrow {\hat{\theta }}_{I}$;

## IV. CRÁMER–RAO LOWER BOUND

In this section, we calculate the Fisher Information Matrix (FIM) and then the Cramér–Rao lower bound (CRLB).^47^ The CRLB provides a lower bound for the variance of an unbiased estimator. The underlying likelihood function (3) contains continuous as well as discrete components so that the conventional method to derive CRLB may not be applicable. A Cramér–Rao type bound for discrete likelihood function is proposed in Ref. 48. However, it cannot handle the distribution containing discrete and continuous components. In this paper, following the calculation in Ref. 6, an approximated CRLB is derived by approximating the discrete component x! by a continuous function xΓ(x). On the other hand, the MLE is typically asymptotically unbiased under mild conditions.^49^ From the simulation given in Sec. V, the proposed MLE asymptotically approaches the derived approximated CRLB.

For the parameter $\theta =[\theta_{1},\theta_{2},\ldots ,\theta_{2m-1},\theta_{2m}]=[M_{1},p_{1},\ldots ,M_{m},p_{m}]$, we see that the (*k*, *l*)th element of the FIM for (2), $I{\left(\theta \right)}_{k,l}$ is

$$I{\left(\theta \right)}_{k,l}=\sum_{i=0}^{M}\sum_{y\in Y_{i}}\left\{\left(\frac{\partial f(y|\theta )}{\partial \theta_{k}}\frac{\partial f(y|\theta )}{\partial \theta_{l}}\right)\frac{1}{f(y|\theta )}\right\},$$ (17)

where $f(y|\theta )=\prod_{j=1}^{m}pr_{j}(y_{j}|M_{j},p_{j})$.

By using x!=xΓ(x) and [xΓ(x)]′=Γ(x)+xΓ(x)ψ(x), where Γ(·) is the Gamma function and ψ(·) is the digamma function, we have

$$\begin{array}{c} \frac{\partial pr_{j}(y_{j}|M_{j},p_{j})}{\partial M_{j}}\\ =\frac{\Gamma (M_{j})p^{y_{j}}{\left(1-p_{j}\right)}^{M_{j}-y_{j}}}{y_{j}!{\left(M_{j}-y_{j}\right)}^{2}\Gamma (M_{j}-y_{j})}\\ \times \{M_{j}(y_{j}-M_{j})\left[\psi (M_{j}-y_{j})-\psi (M_{j})-\text{log}\;(1-p_{j})\right]-y_{j}\} \end{array}$$ (18)

and

$$\frac{\partial f(y_{j}|M_{j},p_{j})}{\partial p_{j}}=-\frac{M_{j}!p_{j}^{y_{j}-1}{\left(1-p_{j}\right)}^{M_{j}-y_{j}-1}(M_{j}p_{j}-y_{j})}{y_{j}!(M_{j}-y_{j})!}.$$ (19)

As a result, by applying the chain rule, we are able to calculate $\left(\partial f(y|\theta )/\partial \theta_{k}\right)$ using (18) and (19). Then, the FIM, I(θ), can now be calculated by inserting (18) and (19) into (17). The CRLB is just $C(\theta )=I{\left(\theta \right)}^{-1}$. For *ν* experiments, the CRLB at ground truth $\theta_{0}$ is $C_{\nu }(\theta_{0})=\left(1/\nu \right)C(\theta ){|}_{\theta =\theta_{0}}$.

## V. SIMULATION

Here, the algorithm is evaluated via Monte Carlo (MC) simulations. The metrics used to evaluate performance are

$$\text{RMSE}({\hat{X}}_{1:\mathrm{nMC},j},X_{j})=\sqrt{\sum_{i=1}^{\mathrm{nMC}}\frac{{\left({\hat{X}}_{i,j}-X_{j}\right)}^{2}}{\mathrm{nMC}}},$$ (20)

$$\text{aMAPE}({\hat{X}}_{1:\mathrm{nMC},1:4},X_{1:4})=\frac{1}{m}\sum_{j=1}^{m}\left(\sum_{i=1}^{\mathrm{nMC}}\left|\frac{{\hat{X}}_{i,j}-X_{j}}{X_{j}}\right|\right),$$ (21)

where $X\in \{M,P\},\;{\hat{X}}_{i,j}$ is the estimate of *X_j_* at the *i*th MC simulation, *nMC* is the number of MC simulations, and RMSE(·) is the root mean square error and aMAPE(·) is the averaged mean absolute percentage error.

To explore the convergence of our algorithm, in Secs. IV A, IV B, and IV C, we illustrate the performance for the one, two, and three distinct species, respectively.

### A. One species

The case of determining the number and detection probability for one species is highly analogous to the case that we presented in Ref. 6. We concentrate on the small number regime as this is more pertinent for the case that we are concentrating on with few emitters.

**Fig. 3.**
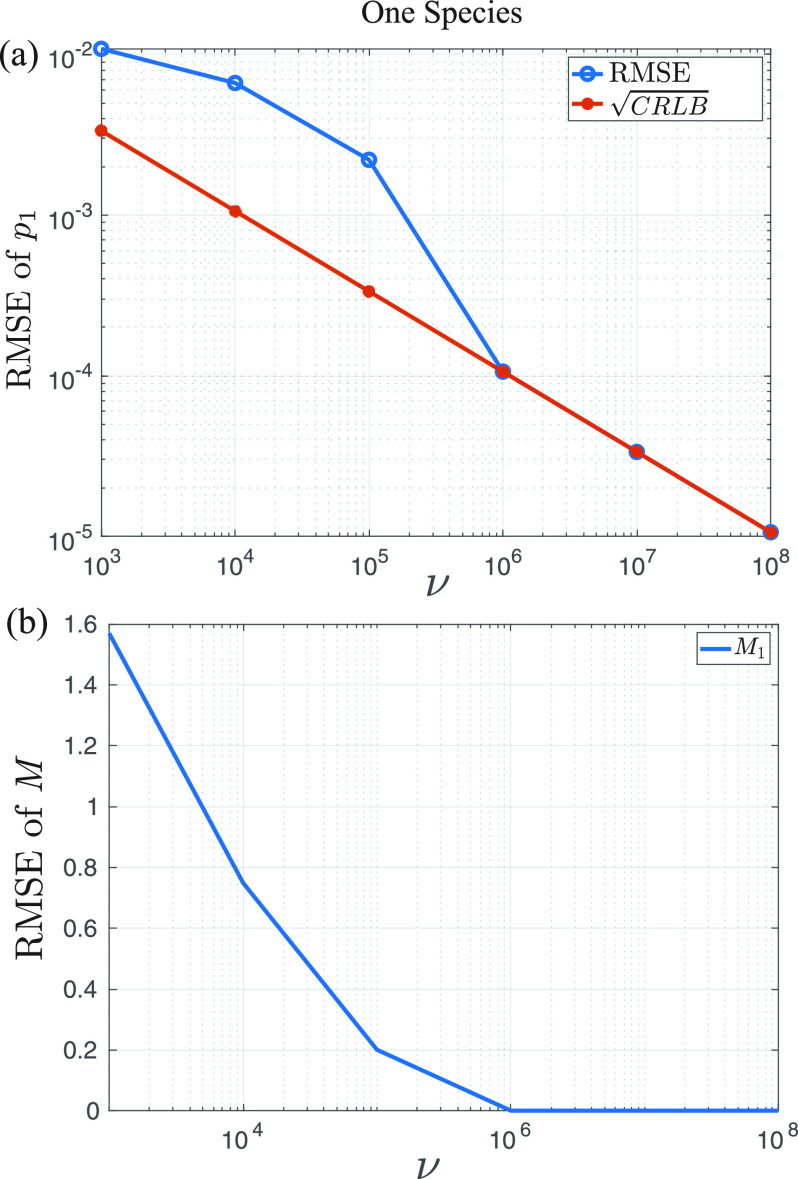
(a) RMSE of the estimate of *p*_1_ as a function of the number of experiments *ν* for a single species. The blue line is the Monte Carlo simulation, and the red is the square root of the CRLB. Good agreement is achieved at around 10^6^ experiments. (b) RMSE of the estimate of the number of emitters as a function of the number of experiments. The ground truth for this case was [M,p]=[8,0.1].

Here, we assume that the true parameters are $\left[M_{1},p_{1}]=[8,0.1\right]$. Figure 3 shows the convergence of the determination of the number of emitters and detection probability.

**Fig. 4.**
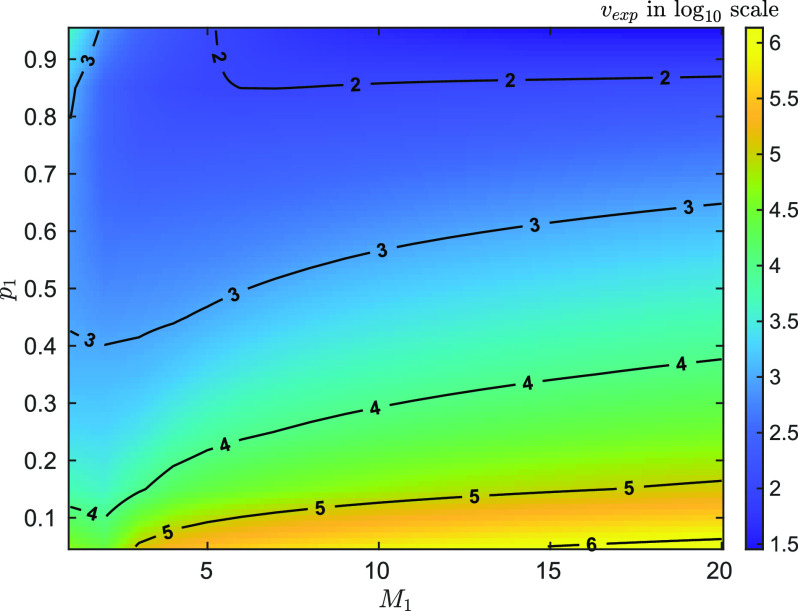
The required number of experiments, *ν_exp_*, to attain $\text{CRLB}(M_{1})/M_{1}=1\%$ for one species of emitter, as a function of *M* and *p*, while *M*_1_ varies from 1 to 20 and *p*_1_ from 0.05 to 0.95. Note that *ν* is plotted in $\;{\text{log}}_{10}$ scale. From the figure, it can be seen that *p*_1_ has greater impact on the required number of experiments, *ν*, i.e., the larger the *p*_1_ is, the less *ν* is. This is because smaller *ν* is required for constructing an empirical distribution $\left[C_{1},\ldots ,C_{N}\right]$ with certain accuracy when *p*_1_ is large. This can be seen from the definition of *p*_1_ and *M*_1_ in (1), where they are parameters of a binomial distribution.

Additionally, in Fig. 4, the (expected) required number of experiments, *ν_exp_*, to attain a given estimation performance, $\text{CRLB}(M_{1})/M_{1}=1\%$, with varied $M_{1}=1,\ldots ,20$ and $p_{1}\in [0.05,0.95]$ is plotted. It can be seen that, to achieve the fixed performance, the small value of *M*_1_ or large value of *p*_1_ requires less number of experiments, which gives some insight in how the values of parameters relate to the estimation performance.

### B. Two species

**Fig. 5.**
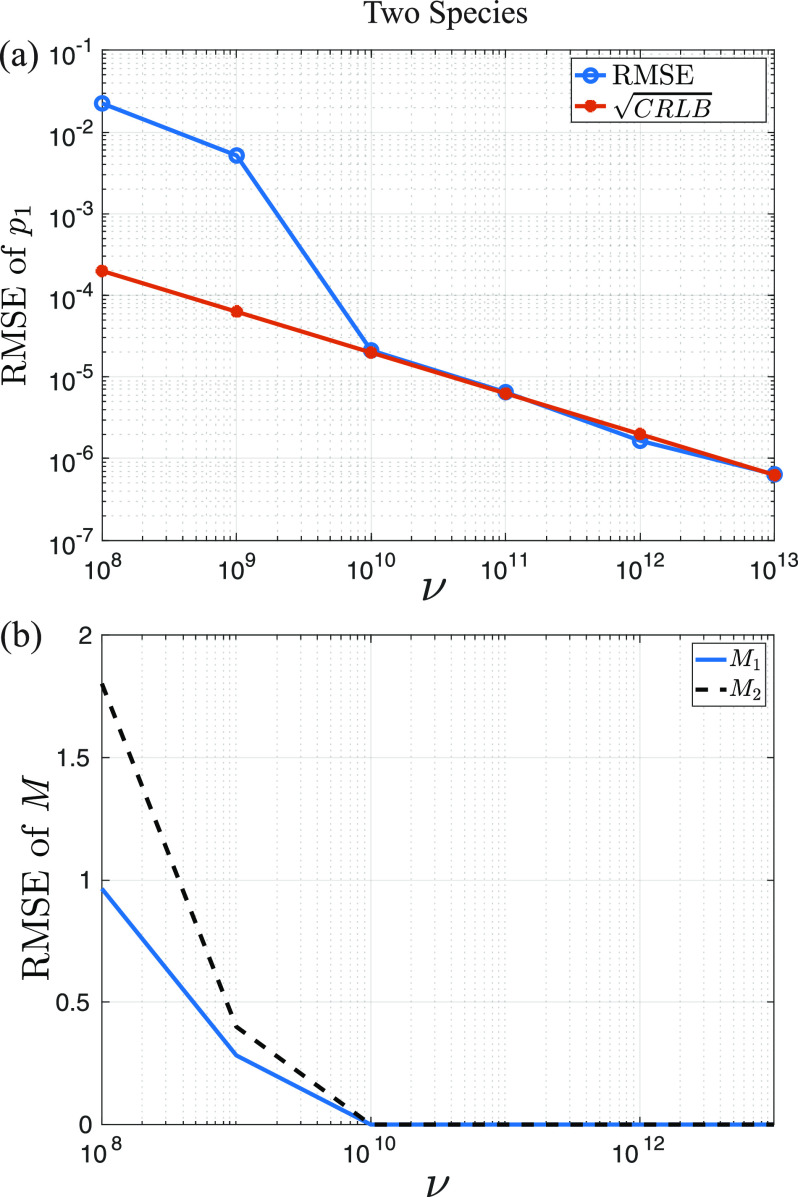
Two species estimation: (a) Reduction in the RMSE of *p*_1_ as a function of *ν* for the simultaneous estimation of the parameters of both species. We omit the results for *p*_2_ as they are very similar to those of *p*_1_. The blue line shows the results of our Monte Carlo simulations, and the red shows the square root of the CRLB. Convergence between the two is achieved at $\nu \approx 10^{10}$ experiments. (b) RMSE of the estimates of *M*_1_ and *M*_2_ as a function of *ν*. Both estimates follow similar trends, with *M*_2_ with the RMSE of *M*_2_ around 1.8 times larger than *M*_1_, due to the fact that it is generally harder to discriminate between states of more emitters.

We now consider the case of two species. As before, we set $\left[M_{1},p_{1}]=[8,0.1\right]$ and introduce $\left[M_{2},p_{2}]=[10,0.2\right]$ as the ground truth condition for the second species. We vary the number of experiments *ν* and examine the reduction in the RMSE of these parameters as *ν* increases, as shown in Fig. 5. Note that our simulations estimate both species simultaneously. For each *ν*, the Monte Carlo simulation is implemented 100 times. The comparison of the simulated RMSE and the computed squared root of the CRLB of *p*_1_ for all *ν* is shown in Fig. 5(a) (the trends for *p*_2_ are not shown since they are similar to *p*_1_), while the comparison of the estimates and the RMSE of the estimated $\left[M_{1},M_{2}\right]$ is shown in Fig. 5(b) for both species.

**Fig. 6.**
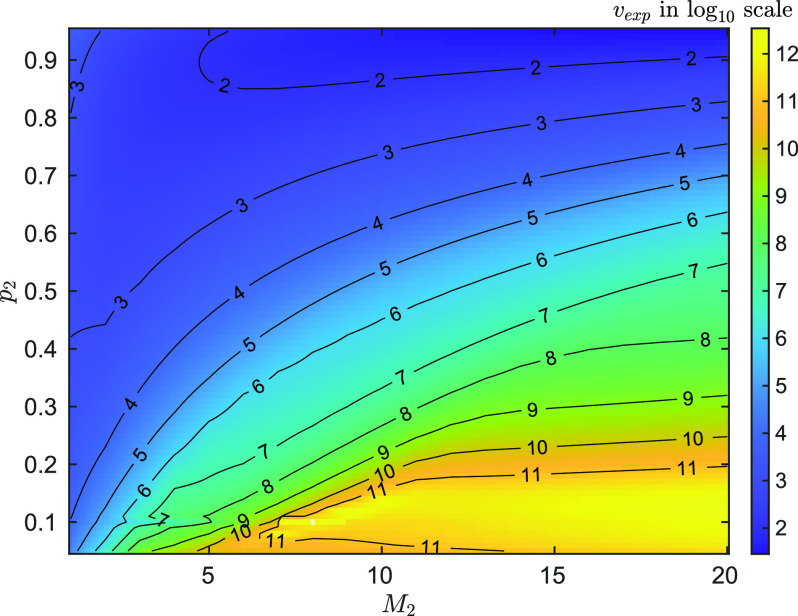
The required number of experiments, *ν_exp_*, to attain $\text{CRLB}(M_{2})/M_{2}=1\%$ with (unknown) $\left[M_{1},p_{1}]=[8,0.1\right]$ as *M*_2_ varies from 1 to 20 and *p*_2_ from 0.05 to 0.95. Note that *ν* is plotted in $\;{\text{log}}_{10}$ scale and the white pixel corresponds to the point that $M_{1}=M_{2}=8$ and $p_{1}=p_{2}=0.1$ so that the FIM is singular (the CRLB does not exist). A similar trend is observed to that shown in Fig. 4, albeit with more experiments required to reach the specified precision for all conditions.

The number of experiments required to obtain $\text{CRLB}(M_{1})/M_{1}=1\%$ and $\text{CRLB}(M_{2})/M_{2}=1\%$ as a function of *M*_2_ and *p*_2_ is shown in Fig. 6, for fixed $\left[M_{1},p_{1}]=[8,0.1\right]$. Though $\left[M_{1},p_{1}\right]$ are fixed, they are treated as unknown parameter in the simulation and estimated simultaneously with $\left[M_{2},p_{2}\right]$. The trends here are highly analogous to those from Fig. 4, albeit at a larger number of experiments for the specified precision. These results show that typically fewer experiments are required for lower *M*_2_, except where *p*_2_ approaches 1. Although the high *p*_2_ limit is not expected to be achievable in standard microscopy type setups.

### C. Three species

**Fig. 7.**
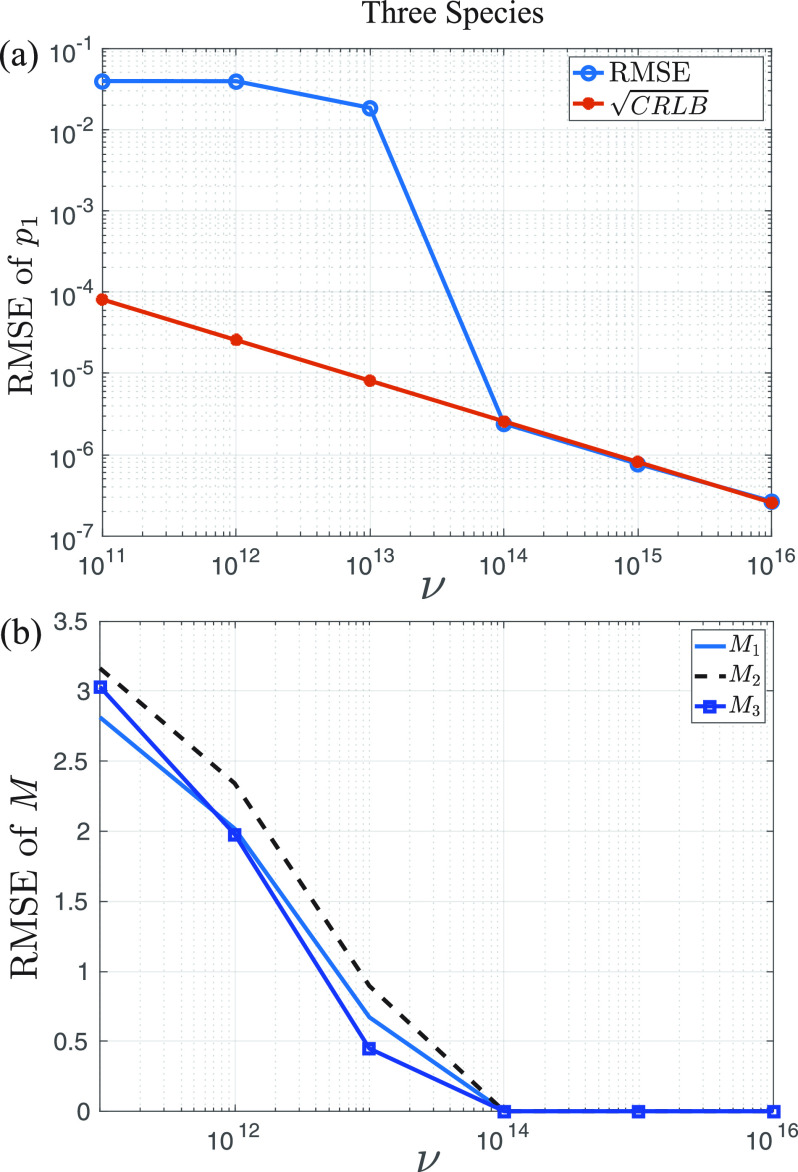
Simultaneous estimation of three species with ground truth $\left[M_{1},p_{1}]=[8,0.1],\;[M_{2},p_{2}]=[10,0.2\right]$, and $\left[M_{3},p_{3}]=[12,0.3\right]$ (a) decrease in the RMSE of *p*_1_ as a function of *ν*. We omit *p*_2_ and *p*_3_ as they are very similar. Convergence with the CRLB is achieved around $\nu =10^{14}$. RMSE for the estimate of the *p_i_* require around three orders of magnitude more experiments before they are similar to those obtained for the two-species estimation due to the complexity of the problem and difficulty in distinguishing the distributions. (b) RMSE of the estimates of *M*_1_, *M*_2_, and *M*_3_ as a function of the number of experiments. Estimates of *M*_2_ are slightly larger than for *M*_1_ and *M*_3_.

We now illustrate our general approach by considering the simultaneous determination of the properties of three species. As before, we maintain the same ground truth solutions for species 1 and 2, such that $\left[M_{1},p_{1}]=[8,0.1],\;[M_{2},p_{2}]=[10,0.2\right]$ and introduce $\left[M_{3},p_{3}]=[12,0.3\right]$. As before we explore the estimation as a function of the number of experiments, with 100 Monte Carlo simulations per experiment (i.e., for each value of *ν*). Convergence of the *p* is similar for all species, so we only show the RMSE of *p*_1_ as a function of *ν*, which is plotted in Fig. 7(a). The overall trends here are similar to those observed with single species and two species identification, although the three species estimation requires around 1000 times more experiments to achieve comparable RMSE in the *p* than the two-species estimation, with convergence to the square root of the CRLB occurring after around 10^14^ experiments, four orders of magnitude more than the two-species convergence.

We show the estimation of *M*_1_, *M*_2_, and *M*_3_ for the same ground truth in Fig. 7(b). These follow similar trends, with the estimation of *M*_2_ being slightly worse than *M*_1_ or *M*_3_ for the same number of experiments. The results show that the estimation requires approximately four orders of magnitude more experiments to achieve similar precision as two-species estimation.

Due to the increasing complexity with increasing number of species, we were unable to simulate the case for more than three species, nor to explore the state space for three-species in the form of Figs. 4 and 6

**Fig. 8.**
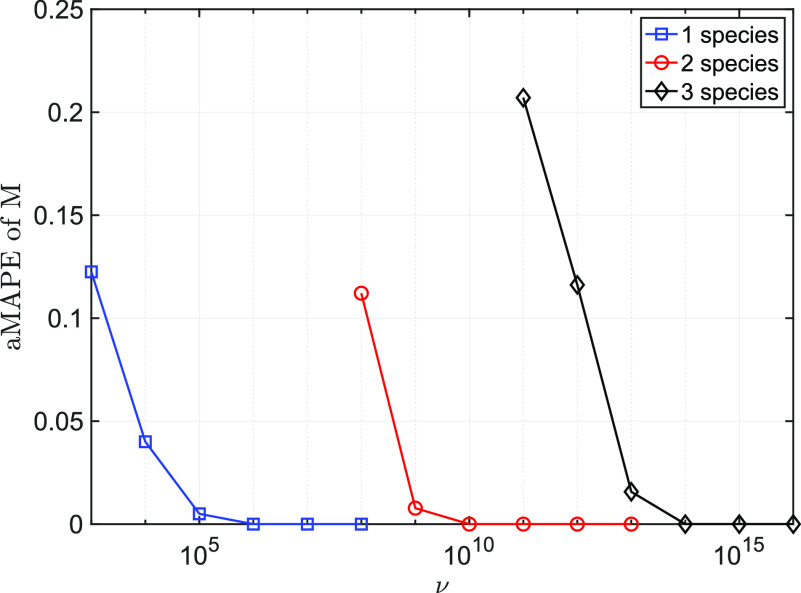
Comparison of the averaged mean absolute percentage errors as a function of *ν* for simultaneous estimation of one species (*M*_1_ only) (blue), two species (*M*_1_ and *M*_2_) (red) and all three species (black). Ground truths were $\left[M_{1},p_{1}]=[8,0.1],\;[M_{2},p_{2}]=[10,0.2\right]$, and $\left[M_{3},p_{3}]=[12,0.3\right]$. Increasing the number of species by one leads to approximately four orders of magnitude in the number of experiments required to obtain similar aMAPE.

Comparing with Fig. 3, Figs. 5 and 7, it is noticeable that the required *ν* for *p*_1_ to attain CRLB increases rapidly with the increasing of *m*, i.e., $\nu \approx 10^{6}$ for *m* = 1, $\nu \approx 10^{10}$ for *m* = 2 and $\nu \approx 10^{14}$ for *m* = 3. Furthermore, the aMAPEs of $\left[{\hat{M}}_{1},\ldots ,{\hat{M}}_{m}\right]$ [see Eq. (21)] are plotted in Fig. 8, where $\left[M_{1},p_{1}]=[8,0.1],\;[M_{2},p_{2}]=[10,0.2\right]$, and $\left[M_{3},p_{3}]=[12,0.3\right]$. One can see, from Figs. 3, 5, 7, and 8, that the performance of estimating *p_j_* and *M_j_* is correlated, with roughly four orders of magnitude more experiments needed as the number of simultaneous species are estimated.

It can be seen from the simulation result, to achieve a certain value of $\left[\text{CRLB}(M_{1})/M_{1}\right]$, the required number of experiments is significantly large, roughly 10^12^, when the probabilities to be estimated are small. This is largely because of that when the probabilities, *p_i_*, are small, the empirical distribution $\left[C_{0},\ldots ,C_{N}\right]$ will be hard to obtain with certain accuracy via limited experiments. As a result, achieving level 1% for $\left[\text{CRLB}(M_{1})/M_{1}\right]$ requires a large number of experiments. It is possible that these results will be improved if *a priori* information is accessible, and so these results should be considered worst case scenarios.

## VI. CONCLUSION

In this paper, we have formulated, mathematically, the estimation of the parameters of an arbitrary number of fluorescent species. Specifically, the convolution binomial model is presented for the underlying problem and then the exact MLE is derived. In order to resolve the intractability of the MLE manifest in the convolutional property and the high-dimensionality of the parameters, a version of the EM algorithm incorporating the method of moment for choosing the initial guess is proposed. The simulation results with different number of species have demonstrated the efficiency of the algorithm by comparing with the derived CRLB.

We also found that the values of the parameters, the number of emitters and their probabilities, have impact on the performance of the estimation: the closer the probabilities are, the worse the performance is, and a larger summed numbers of emitters degrades the performance. Although at present we have assumed that the number of species is known *a priori*, future work could compare models, for example, via the Akaike Information Criterion,^50^ to ease this restriction.

Our work provides a preliminary study and demonstrates the possibility for the estimation of an arbitrary number of fluorescent species and gives insights into the relationship between performance of the estimator and the values of the parameters, which improves understanding of the problem. In future work, we will seek (1) to reduce the required number of experiments while maintaining estimator performance, (2) to improve computational efficiency, (3) to introduce experimental noise signatures, and (4) to identify the role which prior information may help to constrain the estimation problem in particular situations of interest.

## Data Availability

The data that support the findings of this study are available from the corresponding author upon reasonable request.
